# Proteochemometric modelling coupled to *in silico* target prediction: an integrated approach for the simultaneous prediction of polypharmacology and binding affinity/potency of small molecules

**DOI:** 10.1186/s13321-015-0063-9

**Published:** 2015-04-15

**Authors:** Shardul Paricharak, Isidro Cortés-Ciriano, Adriaan P IJzerman, Thérèse E Malliavin, Andreas Bender

**Affiliations:** Department of Chemistry, Centre for Molecular Science Informatics, University of Cambridge, Lensfield Road, CB2 1EW Cambridge, UK; Division of Medicinal Chemistry, Leiden Academic Centre for Drug Research, Leiden University, P.O. Box 9502, , 2300 RA Leiden, The Netherlands; Unité de Bioinformatique Structurale, Institut Pasteur and CNRS UMR 3825, Structural Biology and Chemistry Department, 25-28, rue du Dr. Roux, 75 724 Paris, France

**Keywords:** Target prediction, chemogenomics, proteochemometrics, QSAR, DHFR, plasmodium falciparum

## Abstract

The rampant increase of public bioactivity databases has fostered the development of computational chemogenomics methodologies to evaluate potential ligand-target interactions (polypharmacology) both in a qualitative and quantitative way. Bayesian target prediction algorithms predict the probability of an interaction between a compound and a panel of targets, thus assessing compound polypharmacology qualitatively, whereas structure-activity relationship techniques are able to provide quantitative bioactivity predictions. We propose an integrated drug discovery pipeline combining *in silico* target prediction and proteochemometric modelling (PCM) for the respective prediction of compound polypharmacology and potency/affinity. The proposed pipeline was evaluated on the retrospective discovery of *Plasmodium falciparum* DHFR inhibitors. The qualitative *in silico* target prediction model comprised 553,084 ligand-target associations (a total of 262,174 compounds), covering 3,481 protein targets and used protein domain annotations to extrapolate predictions across species. The prediction of bioactivities for plasmodial DHFR led to a recall value of 79% and a precision of 100%, where the latter high value arises from the structural similarity of plasmodial DHFR inhibitors and *T. gondii* DHFR inhibitors in the training set. Quantitative PCM models were then trained on a dataset comprising 20 eukaryotic, protozoan and bacterial DHFR sequences, and 1,505 distinct compounds (in total 3,099 data points). The most predictive PCM model exhibited *R*^2^_*0*__test_ and RMSE_test_ values of 0.79 and 0.59 pIC_50_ units respectively, which was shown to outperform models based exclusively on compound (*R*^2^_*0*__test_/RMSE_test_ = 0.63/0.78) and target information (*R*^2^_*0*__test_/RMSE_test_ = 0.09/1.22), as well as inductive transfer knowledge between targets, with respective *R*^2^_*0*__test_ and RMSE_test_ values of 0.76 and 0.63 pIC_50_ units. Finally, both methods were integrated to predict the protein targets and the potency on plasmodial DHFR for the GSK TCAMS dataset, which comprises 13,533 compounds displaying strong anti-malarial activity. 534 of those compounds were identified as DHFR inhibitors by the target prediction algorithm, while the PCM algorithm identified 25 compounds, and 23 compounds (predicted pIC_50_ > 7) were identified by both methods. Overall, this integrated approach simultaneously provides target and potency/affinity predictions for small molecules.

**Electronic supplementary material:**

The online version of this article (doi:10.1186/s13321-015-0063-9) contains supplementary material, which is available to authorized users.

**Graphical abstract:**
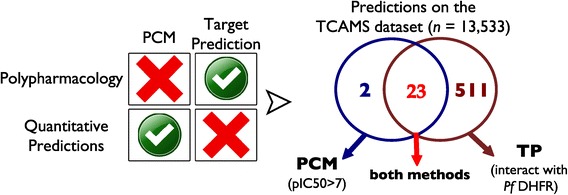
Proteochemometric modelling coupled to in silico target prediction.

## Background

In recent years it has been demonstrated that drugs exert their therapeutic effect by modulating more than one target, in fact six on average [1]. Therefore, the early evaluation of the bioactivity profiles of lead compounds is essential for the success in developing new drugs, although efficacy is sometimes attained by the inhibition of single targets, *e.g.* viral proteins. Similarly, understanding drug polypharmacology can help in anticipating drug adverse effects [2].

In parallel, the availability of public bioactivity databases has enabled the application of large-scale chemogenomics techniques to, among others, predict protein targets for small molecules, and to predict their affinity on therapeutically interesting targets [3]. These techniques capitalize on bioactivity data to infer relationships between the compounds, encoded with numerical descriptors, and their targets, which can be represented as labels in a classification model or explicitly encoded by *e.g.* protein or amino acid descriptors [4].

*In silico* target prediction algorithms assess potential compound polypharmacology through the computational evaluation of the (functionally unrelated) targets modulated by a given compound, or its selectivity to species-specific targets, as they predict the probability of interaction of that compound with a panel of targets [5]. Initially, target prediction models were developed using Laplacian-modified Naïve Bayesian classifiers [6] and the Winnow algorithm [7]*.* Later, Keiser *et al.* [8] developed a model which related biological targets based on ligand similarities and ranked the significance of the resulting similarity scores using the Similarity Ensemble Approach (SEA), followed by Wale and Karypis [9] who applied SVM and ranking perceptron algorithms to rank targets for a given compound. More recently, Koutsoukas *et al.* [10] compared the performance of both the Naïve Bayesian and Parzen-Rosenblatt Window classifiers, concluding that the overall performance of both methods is comparable though differences were found for certain target classes.

The ligand-target prediction methods described above generally predict the likelihood of interaction with a target, and they do not predict compound affinity or potency (*e.g.* K_i_ or IC_50_). On the other hand, quantitative bioactivity prediction techniques, *e.g.* proteochemometric modelling (PCM) [3], predict the potency or affinity for compound-target pairs, normally in the form of pIC_50_ or pK_i_ values. PCM combines information from compounds and related targets, *e.g.* orthologs, in a single machine learning model [3,11], which enables the simultaneous modelling of chemical and biological information, and thus the prediction of compound affinity and selectivity across a panel of targets. Nonetheless, the effects of a compound at the cellular or the organism level are poorly understood in this case, as these methods cannot account for the interactions of a compound with other unrelated targets, which are not captured in the PCM model.

Given the limitations of both purely qualitative and purely quantitative bioactivity modelling approaches, in the current work, we propose an integrated drug discovery approach, combining *in silico* target prediction for the qualitative large-scale evaluation of compound bioactivity, and PCM for the quantitative prediction of compound potency. The proposed approach was evaluated on the discovery of DHFR inhibitors for *Plasmodium falciparum* (*P. falciparum*), the causative agent of the most dangerous form of malaria [12]. Whilst there are multiple anti-malarial drugs on the market, resistance to anti-malarial drugs is on the rise [13,14], and there are only 21 compounds in clinical or pre-clinical trials [15].

In order to combat the lack of novel drugs for malaria, big pharmaceutical companies have generated a wealth of phenotypic data, namely the GlaxoSmithKline (GSK) TCAMS dataset, as well as the Novartis-GNF Malaria Box [16,17]. Both datasets contain phenotypic readouts, describing how effective the compounds present in the datasets are in inhibiting the growth of *P. falciparum.* Nonetheless, none of them contain annotations about the *P. falciparum* target(s) involved, making it a challenge to elucidate the mode of action (MoA) of the compounds in the dataset, and hence, making the dataset difficult to interpret. This renders these datasets a very suitable case study for the algorithms we are presenting in this work.

In the context of malaria drug discovery, previous studies have applied machine learning algorithms to predict whether plasmodial proteins are secretory proteins based on their residue composition [18], and to predict the bioactivities of compounds against particular plasmodial targets [19,20]. These approaches, though, did not account for the polypharmacology of anti-malarial compounds.

**Figure 1:**
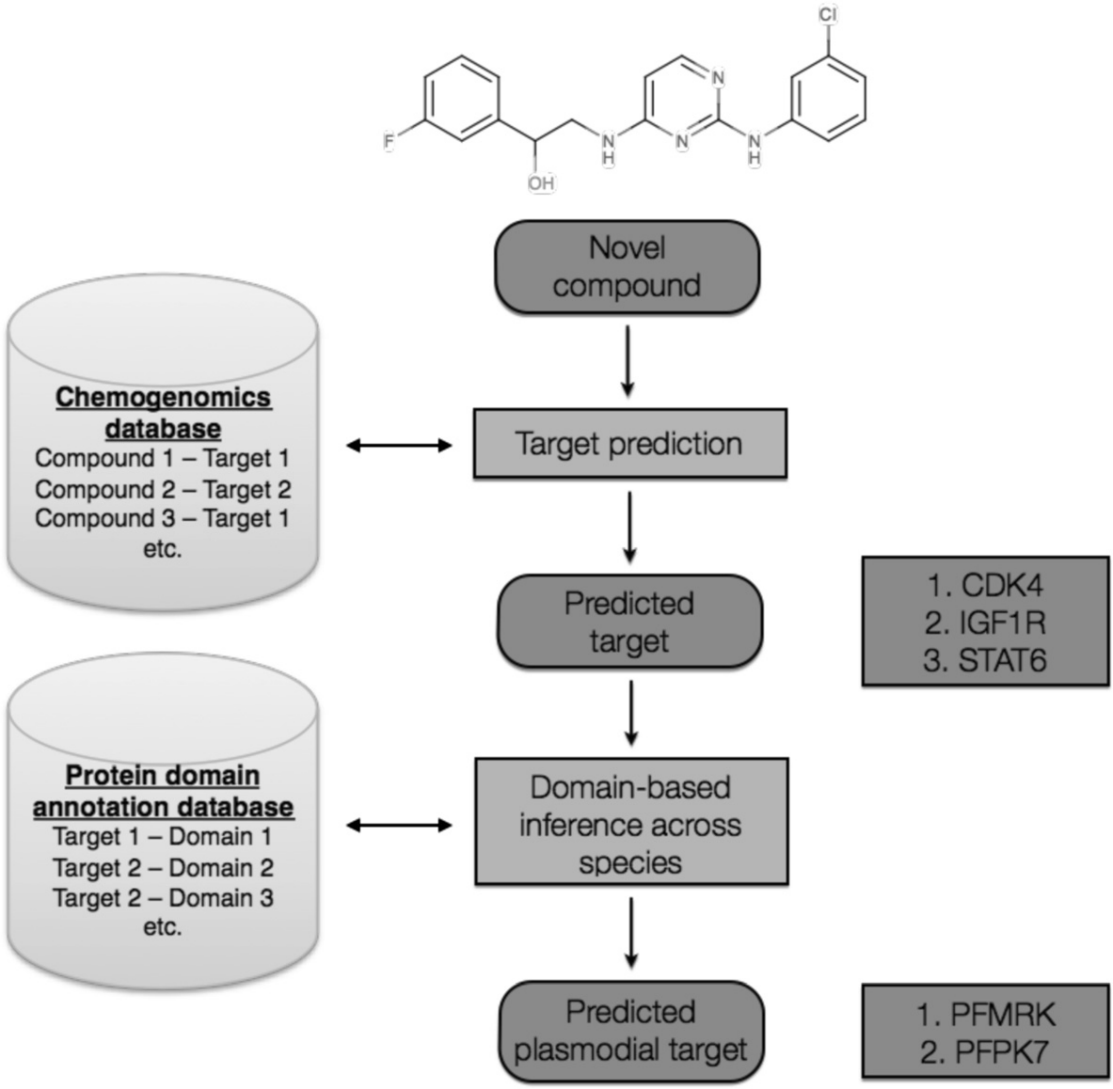
Schematic overview of *in silico* target prediction and domain-based extrapolation workflow. The conventional *in silico* target prediction approach [10] is extended in this study by using protein domain annotations to extrapolate from non-plasmodial target predictions to protein target predictions in *P. falciparum.* This concept is generally applicable across organisms, in particular to those for which little bioactivity data is currently available.

To overcome the limitations of these methods, we now integrate both *in silico* target prediction and PCM in a unified drug discovery approach. As illustrated in Figure 1, the target prediction algorithm used in this study, trained on approximately 553,084 bioactivity data points spanning 3,481 targets, used a domain-based similarity metric between targets to extrapolate target predictions from one species to another. Non-plasmodial targets were then extrapolated to plasmodial targets. Besides, the PCM model was trained on a dataset composed of 20 eukaryotic, protozoan and bacterial DHFR sequences, and of 1,505 different DHFR inhibitors and a total of 3,099 data points. To exploit the complementarity of the two prediction methods, *in silico* target prediction was used to predict MoA hypotheses for the anti-malarial compounds in the GSK TCAMS phenotypic dataset, whereas PCM was employed to quantify compound potency (pIC_50_).

## Methods

### Exploratory principal component analysis (PCA) of PCM and target prediction datasets

A PCA was performed for compounds contained in the PCM dataset, as well as for those annotated on *P. falciparum* and *T. gondii* in the target prediction dataset. The Spearman’s rank correlation coefficient was calculated for all pairs of compound descriptors, based on both physicochemical descriptors and Morgan fingerprints, thus defining a square correlation matrix. The PCA analysis was performed on this matrix in order to avoid the direct application of PCA on binary descriptors, *i.e*. Morgan fingerprints. Visualization was performed using R and Vortex [21].

### Target prediction

#### Training dataset

Bioactivity data were extracted from ChEMBL16 [22] according to the protocol described by Koutsoukas *et al.* [10]. The extracted data contained approximately 4 million bioactivities covering approximately 8,000 biomolecular targets, of which approximately 4,000 targets were proteins [22,23]. Compound-target pairs were selected according to the following criteria: (i) K_i_, K_d_, IC_50_ or EC_50_ bioactivity values equal to or lower than 10 μM, and (ii) targets annotated with a confidence score of 8 (homologous single protein target assigned) or 9 (direct single protein target assigned). Subsequently, ligand structures were processed with the ChemAxon standardizer version 5.12.0 [24], with the following options: “Remove fragment”, “Neutralize”, “Aromatize”, “Clean2D”, “Tautomerize” and “Remove explicit hydrogens”. After standardization*,* the entries with ligands annotated against multiple targets were detected based on their canonical SMILES and removed using custom Perl scripts, resulting in a training set of 553,084 instances (262,174 compounds) covering 3,481 protein targets (Additional file 1: Supplementary Information SI 1). The bioactivity data of *P. falciparum* (1,513 instances – 1,379 compounds covering 41 protein targets) was omitted from this dataset for training purposes. InterPro [25] domain annotations were retrieved for all protein targets using the Uniprot database [26].

#### *P. falciparum* dataset

The *P. falciparum* dataset was built using the same criteria as described above, resulting in a set comprising 41 *P. falciparum* targets and 1,379 compounds. In addition, all annotated and reviewed *P. falciparum* targets from Uniprot were downloaded, resulting in a total of 148 *P. falciparum* protein targets. Finally, InterPro domain annotations were retrieved for all protein targets using the Uniprot database (Additional file 2: Supplementary Information SI 2).

#### GSK TCAMS dataset

Approximately 2 million compounds present in GSK’s screening collection have been tested *in vitro* by GSK for inhibitors of *P. falciparum’s* intraerythrocytic cycle based on growth inhibition assays [17]. Briefly, assays were performed on both the reference laboratory strain 3D7, as well as on the multidrug resistant strain Dd2, where parasite growth was evaluated using LDH activity [17]. 19,451 compounds were identified as primary hits inhibiting the 3D7 strain growth by more than 80% at 2 μM concentration, of which 13,533 compounds displayed 80% or higher inhibition of parasite growth in at least 2 of the 3 assay runs in independent follow-up experiments. Hence, these 13,533 compounds were considered as confirmed inhibitors (confirmation rate > 70%) and used in the present study.

#### Descriptors

A circular fingerprint implementation, Molprint2D [27,28] was used for encoding molecular structures, since this method has previously been shown to capture structural aspects related to bioactivity better than most other descriptors in comparative studies [29]. This descriptor is based on count vectors of heavy atoms present at a topological distance from each heavy atom of a molecule [28]. For the present study, the pybel implementation was used [30].

### Target prediction algorithm

A multiclass Laplacian-modified Naïve Bayesian classifier, as described by Nigsch *et al.* [7] and later implemented by Koutsoukas *et al*. [10] was implemented to classify the bioactivity dataset and to be able to predict targets for novel compounds. For the query molecule ***x***, consisting of a set of *n* Molprint2D features *f*_*i*_, the likelihood to be active against a protein target *ω*_*α*_ was calculated using the following equation:

1 $$ {S}_{{\boldsymbol{\omega}}_{\boldsymbol{\alpha}}}\left(\boldsymbol{x}\right)={\displaystyle \sum_{\boldsymbol{i}=1}^{\boldsymbol{n}}} log\left(\frac{N_{i,{\omega}_{\alpha }}+1}{N_i\times p\left({\omega}_{\alpha}\right)+1}\right)+ log\left(\frac{{\displaystyle {\prod}_{i=1}^d}p\left({f}_i\right)}{p\left(\boldsymbol{x}\right)}\right) $$

where ${S}_{{\boldsymbol{\omega}}_{\boldsymbol{\alpha}}}\left(\boldsymbol{x}\right)$ is the logarithmic likelihood score (proportional to the likelihood of bioactivity), ${N}_{i,{\omega}_{\alpha }}$ is the total number of occurrences of feature *f*_*i*_ in protein class *ω*_*α*_ and *N*_*i*_ is the total number of occurrences of feature *f*_*i*_ in the entire training set. Furthermore, *p*(*ω*_*α*_) is the prior probability of protein class *ω*_*α*_. The prior probability quantifies how likely a compound is active against protein target *ω*_*α*_ in the absence of any feature information. It can be calculated as follows:

2 $$ p\left({\omega}_{\alpha}\right)=\frac{N_{\omega_{\alpha }}}{N} $$

where ${N}_{\omega_{\alpha }}$ is the number of instances (*i.e.* bioactivities) in class *ω*_*α*_ and *N* is the total number of instances. The predictive performance of this model was assessed in terms of average class-specific recall and precision. Only target classes with 20 or more data points in the *P. falciparum* dataset were considered as suitable for testing due to a sufficient number of data points, resulting in a total of 16 target classes.

#### Domain-based extrapolation to *P. falciparum* targets

For each analyzed compound, the top *n* ranked predicted targets were compared to all 148 *P. falciparum* targets in terms of their InterPro domain composition. *P. falciparum* targets with an InterPro domain Tanimoto similarity above a variable cut-off were considered as predicted, but were not ranked. The cut-off value varied between 0.5 and 1, where 1 means that only orthologous proteins are considered. The target prediction and domain-based extrapolation pipeline are illustrated in Figure 1. The domain extrapolation extends the target prediction approach [10,31] by using InterPro protein domain annotations to extrapolate from predicted non-plasmodial targets to *P. falciparum* targets. This is conceptually similar to a previously reported study for extrapolating bioactivities between species [32], and its application to *M. tuberculosis* [33].

The inclusion of plasmodial DHFR (CHEMBL1939) bioactivity data was expected to drastically improve the performance, and this was tested in the following way. A 2-fold cross validation (CV) was performed: the instances annotated on plasmodial DHFR were split into 2 half subsets, where one subset was added to the training set and the other half was used as a test set (and *vice versa*).

### Proteochemometric modelling

#### Dataset

IC_50_ values with a confidence score of 8 or 9 for 20 DHFR sequences (Table S3) were retrieved from ChEMBL16 [22] and this initial dataset comprised 5,827 data points. In the cases where a compound-target combination had more than one annotated bioactivity value, the set of bioactivities was replaced by its mean value. This procedure is robust, because the standard deviation of the differences was smaller than 0.1 pIC_50_ unit in more than 90% of the cases (Additional file 3: Figure S1). This resulted in a dataset including 3,099 distinct compound-target combinations. The matrix completeness of the dataset, calculated as the number of compound-target combinations present in the dataset over the total number of possible compound-target combinations, was 10.3%. Compounds included in the PCM dataset were not present in the target prediction dataset.

#### Descriptors

Chemical structures were standardized and cleaned with the function *StandardiseMolecules* of the R package *camb* using the default parameters [34] and PaDEL descriptors (1-D and 2-D). Morgan fingerprints were calculated in the same environment. The function *AA_Descs* was used to calculate amino acid descriptors (3 Z-scales). To describe the target space, the residues in the binding site of human DHFR (PDB ID: 1OHJ [35]) within a sphere of 10 Å centered around the ligand were selected. The corresponding residues for the other 19 proteins were obtained from a sequence alignment realized with Clustal Omega [36]. The dataset is available in Additional file 4 (Supplementary Information SI 3).

#### Proteochemometric modelling

All descriptor values were centered to zero mean and scaled to unit variance. The dataset was split into six subsets, five of which were used to train models, and the sixth, test set, was withheld to assess the predictive ability of the models [37]. The hyperparameter values for all PCM models were optimized by 5-fold cross validation [38]. To assess both model predictive ability and performance, the pIC_50_ values for the test set were predicted, thus providing the external validation by calculating RMSE_test_ and *R*^2^_*0*__test_ between the observed and the predicted pIC_50_ values:

3 $$ {R}_{0\  test}^2=1-\frac{{\displaystyle {\sum}_{i=1}^N}{\left({y}_i-{\widehat{y}}_i^{r0}\right)}^2}{{\displaystyle {\sum}_{i=1}^N}{\left({y}_i-\overline{y}\right)}^2} $$

4 $$ RMSE=\sqrt{\frac{{\left(y-\widehat{y}\right)}^2}{N}} $$

where *N* represents the size of the test set, *y*_*i*_ the observed, *ŷ*_*i*,_ the predicted, and $\overline{y}$ the average pIC_50_ values of those datapoints included in the test set, and *ŷ*_*i*_^*ro*^ = *sŷ*, with $s={\displaystyle \sum {y}_i{\widehat{y}}_i}/{\displaystyle \sum {\widehat{y}}_i^2}$. Both internal (RMSE_int_ and *R*^2^_int_) and external validation (RMSE_test_ and *R*^2^_*0*__ext_) were assessed according to the criteria proposed by Tropsha *et al.* [39,40] and calculated using the *Validation* function of the R package *camb* [34]*.*

In order to assess whether the combination of compound and target information in a single PCM model constitutes an advantage with respect to one-space (ligand space and target space) models, two validation scenarios were explored. Firstly, a Family QSAR model [41] was trained exclusively on compound descriptors. High performance of this model is expected in cases where the bioactivities of the same compound on different targets are highly correlated. Secondly, the Family QSAM [41] model was trained on target descriptors only. In this case, high performance would indicate that the activities of a diverse set of compounds are correlated on a panel of targets. Thus, compound activities would largely depend on the target, and to a much lesser extent on the ligand structures.

Additionally, an inductive transfer PCM model (PCM IT) was trained to assess whether the performance of PCM models arises from explicit learning (EL), where the knowledge is extracted from target descriptors, or inductive transfer (IT). In IT the knowledge acquired when predicting compound bioactivities on a given target is exploited to predict the bioactivity of those compounds on another target [41]. In the PCM IT model, targets were described with identity fingerprints (IFP), which are calculated as follows:

5 $$ IFP\left(i,j\right)=\delta \left(i-j\right)\left(i,j\in 1,\dots, {N}_{\mathrm{targets}}\right) $$

where δ is the Kronecker delta function and N_targets_ the number of distinct targets. The performance of the models was assessed on a *per* target basis by training ten PCM models, each on a different subset of the whole dataset. Subsequently, RMSE_test_ and *R*^2^_*0*__test_ values were calculated on subsets of the test set grouped by target.

### Machine learning implementation

Support Vector Machines (SVM) [42], Gradient Boosting Machines (GBM) [43], Gaussian Processes (GP) [44], and Random Forest (RF) [45] models were built with the R package *camb* [34,46]. The target prediction algorithm was implemented in Perl.

## Results and discussion

### Exploratory analysis of PCM and target prediction datasets

**Figure 2:**
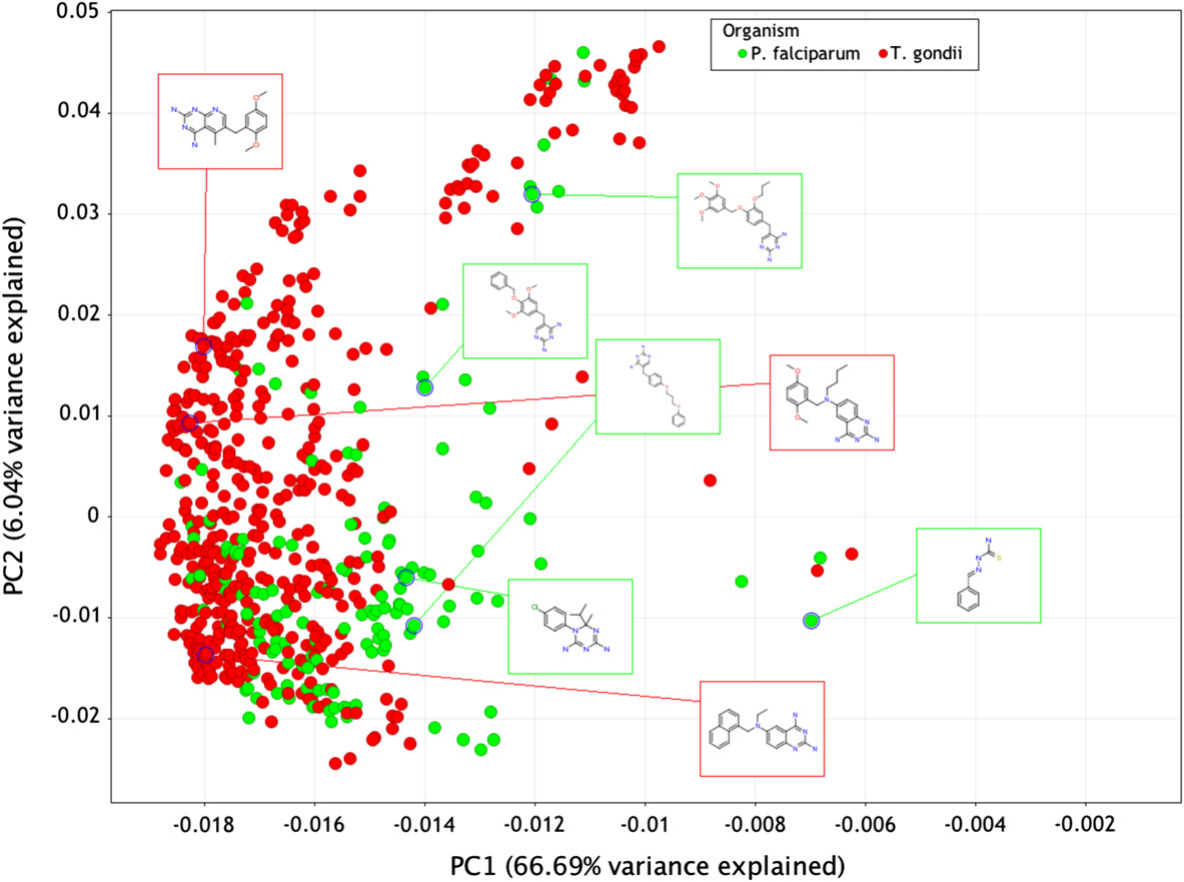
PCA of the compounds annotated as actives against plasmodial DHFR (green) as well as *T. gondii* DHFR (red). Overall, plasmodial DHFR inhibitors cover a substantial portion of the chemical space occupied by *T. gondii* DHFR inhibitors. However, some clusters of *T. gondii* DHFR inhibitors are located in additional chemical space not covered by the plasmodial inhibitors (red boxes). These clusters contain compounds with bicyclic ring systems. By contrast, plasmodial inhibitors only contain unfused rings (green boxes). These observations explain why recall is low (~35%) when plasmodial DHFR inhibitors are excluded from the training set: *T. gondii* inhibitors do not cover all relevant chemical space, particularly the space occupied by compounds with unfused ring systems.

A PCA (Figure 2) was performed for the compounds annotated to be active against plasmodial DHFR and those active against *T. gondii* DHFR. The first two principal components explain 72.73% of the variance. In the two dimensions visualized for the descriptor space used here, the plasmodial inhibitors cover a substantial portion of the chemical space occupied by the *T. gondii* DHFR inhibitors. However, there are still a number of clusters of *T. gondii* DHFR inhibitors that occupy novel space not covered by plasmodial inhibitors. Compounds from these clusters contain bicyclic ring systems (shown in red boxes in Figure 2). On the other hand, there are also clusters of plasmodial inhibitors that occupy space not covered by *T. gondii* inhibitors: these plasmodial inhibitors do not contain bicyclic rings, but instead contain unfused rings (5 scaffolds identified shown in green boxes in Figure 2). In addition to the previous analysis, a PCA was also performed for the compounds present in the PCM dataset (Additional file 3: Figure S2), where the first two principal components explained 51.77% of the variance. Clusters contain compounds whose bioactivities on several targets are included in the dataset, thus indicating that compounds are overall structurally similar across the 20 DHFR sequences considered.

### Application of target prediction for MoA prediction

The performance of the target prediction algorithm was assessed for varying values of *n*, which represents the top number of non-plasmodial predictions considered for extrapolation (Additional file 3: Figure S3). It can be seen that performance varies widely across target classes: for most targets, including all aminopeptidases, calcium-dependent protein kinase 1, protein kinase Pfmrk, glucose-6-phosphate-1-dehydrogenase, dihydroorotate dehydrogenase, dUTP pyrophosphatase and enoyl-acyl-carrier protein reductase, performance is low, with both recall and precision values below 30%. For a small number of targets, however, the performance is much higher, with recall values up to ~60% and precision values up to 100%. Further investigation revealed that the targets for which the prediction algorithm performed well (plasmepsin 1 and 2, histone deacetylase, DHFR and to a lesser extent, falcipain 2) were plasmodial orthologs of non-plasmodial protein targets. This finding is in agreement with previous studies, which have used orthologous proteins to extrapolate the prediction of bioactivities between target classes across species such as *P. falciparum* and *M. tuberculosis* [47,48]. However, these previous studies have not combined target prediction with PCM for MoA analysis, which is precisely the novelty of the approach presented here.

### Target prediction performance for plasmodial DHFR

The predictive performance of the target prediction algorithm was further investigated for the plasmodial target DHFR, where all 145 instances annotated on plasmodial DHFR were used as a test set. The top *n* predicted non-plasmodial targets were considered (*n* varied in the 1–12 range), after which these targets were extrapolated to plasmodial targets (section “Domain-based extrapolation to *P. falciparum* targets” in Materials and Methods). For *n* in the 1–3 range, the recall values are 0%, 2.8% and 14,5%, respectively, whereas for *n* in the 4–7 range, the recall values are around 35%. The 2-fold CV resulted in a recall value of 79%. These results indicate that despite the fact that the training set did not contain any plasmodial bioactivity data, the model is still able to predict compounds active against plasmodial DHFR with 100% precision, based on bioactivity data for orthologous proteins across other species. The high precision value arises from the structural similarity of plasmodial DHFR inhibitors and *T. gondii* DHFR inhibitors in the training set (the average MOLPRINT2D pairwise similarity between the *T. gondii* inhibitors and the plasmodial inhibitors was 16%, whereas the average pairwise similarity within the plasmodial dataset and the *T. gondii* dataset was 19% and 18% respectively). These results show the added benefit of incorporating domain-based extrapolation for target prediction purposes.

In addition, we found that varying the domain Tanimoto similarity cut-off between 0.5 and 1 did not alter the performance. Hence, in order to maintain high precision, a stringent domain Tanimoto similarity cut-off of 1 (*i.e.* requiring a 100% overlap in domain presence and absence between two proteins) was chosen and the top *n* predicted non-plasmodial targets considered was set to 4 for further analysis. Further investigation of the extrapolation from non-plasmodial targets to plasmodial targets revealed that only one protein class (*T. gondii* DHFR) was responsible for the extrapolation of predicted activities to plasmodial DHFR. As described earlier, there are clusters of *T. gondii* DHFR inhibitors that do not contain any plasmodial DHFR inhibitors (scaffolds identified in these clusters are shown in red boxes - Figure 2 and clusters of plasmodial inhibitors that occupy space not covered by *T. gondii* inhibitors (5 scaffolds identified shown in green boxes in Figure 2). Hence, for these clusters there is no overlap in scaffolds between both datasets. These observations explain the low recall of the model at this stage: plasmodial DHFR inhibitors located outside the space covered by *T. gondii* DHFR inhibitors are not retrieved by the model, thereby increasing the number of false negatives, whereas the plasmodial DHFR inhibitors that are present in the chemical space shared by inhibitors from both species are predicted with very high precision.

**Figure 3:**
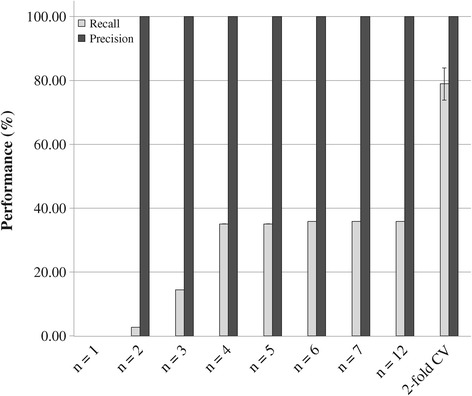
Performance of the DHFR target prediction model compared across a number of parameters. 145 data points annotated against plasmodial DHFR were used as a test set to assess the performance of the target prediction model. The top *n* predicted non-plasmodial targets were considered (*n* was varied for values between 1 and 12), after which these targets were extrapolated to plasmodial targets. When *n* increases, recall values rise up to 36% (with recall values of ~35% for n =3 and n = 4). On the other hand, precision values are 100% for *n* ≥ 2. The high precision values are likely to be explained by the fact that plasmodial DHFR inhibitors and *T. gondii* DHFR inhibitors occupy the same chemical space. In addition to varying the parameter *n*, we performed a 2-fold cross validation (averaged over 20 randomizations), which resulted in a drastic improvement as a recall value of 79% was achieved (with a standard deviation of 10.1%, which is shown as an error bar). These results show that domain-based extrapolations have added value to the prediction algorithm (correct predictions are made even when bioactivity data on plasmodial DHFR is not present in the training set) and that including plasmodial DHFR bioactivity data in the training set can drastically improve recall values.

Adding plasmodial DHFR data to the training set drastically increased performance, more than doubling recall values to 79%, whereas precision values remained 100% (Figure 3 – 2-fold CV). Hence, this observation arises from the fact that the chemical space of the plasmodial DHFR inhibitors adds additional information corresponding to 5 new scaffolds (as highlighted in green boxes in Figure 2) to the model. However, despite the very high precision value achieved (100%), there is a drawback: given the great increase in recall value when novel scaffolds are added to the dataset, the model is only able to correctly predict bioactivities for compounds with scaffolds that are already present in the training data. Hence, a diverse set of molecules is required in the training set in order to optimize recall values of the model. Given the benefit of both domain-based extrapolation and using plasmodial DHFR bioactivity data for model training, all plasmodial DHFR data were included in the training set for further MoA prediction of the GSK TCAMS phenotypic dataset in order to optimize recall values.

### PCM model validation

The four algorithms used in this study (GBM, GP, RF and SVM) displayed similar performance on this dataset as the ranges of RMSE_test_ and R^2^_0 test_ differences are 0.04 pIC_50_ and 0.02 units, respectively. The GBM model exhibited the highest predictive ability with R^2^_0 test_ and RMSE_test_ values of 0.79 and 0.59 pIC_50_ units respectively. Both internal and external validation metrics are given in Table 1.

**Table 1:** **PCM, Family QSAR and Family QSAM performance on the PCM dataset**

	**R** ^**2**^_**CV**_	**RMSE**_**CV**_	**R** ^**2**^_**0 ext**_	**RMSE**_**ext**_
GBM PCM	0.75	0.64	0.79	0.59
GP PCM	0.75	0.65	0.76	0.63
RF PCM	0.74	0.66	0.77	0.62
SVM PCM	0.76	0.63	0.77	0.62
Family QSAM	0.07	1.24	0.09	1.22
Family QSAR	0.61	0.80	0.63	0.78
Inductive Transfer	0.72	0.68	0.76	0.63

Abbreviations: *QSAM* Quantitative Structure-Activity Modelling, *QSAR* Quantitative Structure-Activity Relationship, *GBM* Gradient Boosting Machine, *GP* Gaussian Process, *RF* Random Forest, *SVM* Support Vector Machine.

PCM, with R^2^_0 test_ and RMSE_test_ values of 0.79 and 0.59 pIC_50_ units, outperforms both Family QSAR, with R^2^_0 test_ and RMSE_test_ values of 0.63 and 0.78 pIC_50_ units, respectively, and Family QSAM, with with R^2^_0 test_ and RMSE_test_ values of 0.09 and 1.22 pIC_50_ units, respectively.

To ensure that the model’s predictive ability was not the consequence of spurious correlations in the data, we trained ten GBM models with an increasingly higher percentage of the pIC_50_ values randomized. Additional file 3: Figure S4 shows the performance of the ten models, quantified by the RMSE_test_ and R^2^_0 test_ values as a function of the level of randomization of the bioactivity values. The intercept was zero or negative when ~40% of the response variable was randomized (Additional file 3: Figure S4A). Therefore, the relationship established by the PCM models between the descriptor space and the bioactivity values is not a consequence of chance correlations [49].

### PCM outperforms one-space models and IT on this dataset

The Family QSAM model, trained on target descriptors only, displayed poor predictive ability with RMSE_test_ and R^2^_0 test_ values of 1.22 pIC_50_ units and 0.09, respectively (Table 1). By contrast, the Family QSAR model, trained on compound descriptors only, displayed satisfactory values for the statistical metrics according to our validation criteria, as the model exhibited RMSE_test_ and R^2^_0 test_ values of 0.78 pIC_50_ units and 0.63, respectively (Table 1). Hence, compound descriptors explain a large proportion of the variance, which may stem from the high correlation of the bioactivities of identical compounds against orthologs. Indeed, Additional file 3: Figure S5 depicts the correlation (RMSE: 0.95 pIC_50_ units; R^2^_0_: 0.46) between the pIC_50_ values of the same compounds on different orthologs.

Furthermore, better performance is obtained for the GBM PCM model trained on amino acid descriptors and compound fingerprints, than for the GBM model trained on target identity fingerprints and compound fingeprints, with RMSE_test_ values of 0.59 vs. 0.63 pIC_50_ units, respectively. This indicates that our selection of amino acid descriptors captured the binding site information of the different orthologs and thus allows explicit learning on this dataset (Table 1). Overall, these data suggest that the explicit inclusion of target information improves bioactivity prediction.

### Several high-affinity DHFR Inhibitors are identified by both target prediction and PCM

The targets for which the target prediction model had a class-specific F-measure higher than 40% were selected, leading to a shortlist of 5 proteins, namely: plasmepsin 1 and 2, histone deacetylase, DHFR and falcipain 2 (Additional file 3: Figure S6). Overall, a total of 1,291 plasmodial predictions were made for 1,017 compounds. DHFR is the most commonly predicted target, which represents 534 (41%) of the total predictions, followed by plasmepsin 1 (280 predictions – 22%) and plasmepsin 2 (273 predictions – 21 histone deacetylase (184 predictions – 14%) and falcipain 2 (20 predictions – 2%). Plasmodial DHFR has previously been proposed as a candidate target against resistant plasmodial strains [50]. In addition, the plasmepsin (1 and 2) and falcipain targets have previously been proposed as potential targets for anti-malarial therapy [51], due to their involvement in the hemoglobin catabolism that occurs during the erythrocytic stage of the malarial parasite life cycle (plasmepsin proteins and falcipain proteins), and to their involvement in erythrocyte invasion and erythrocyte rupture (falcipain proteins) [52]. Finally, plasmodial histone deacetylase has been proposed as a promising target for anti-malarial therapy due to its key role in regulating gene transcription, and it has been shown that histone deacetylase inhibitors are potent inhibitors of the growth of *P. falciparum* [53]. Hence, there is sufficient evidence for all 5 predicted proteins for being a potential target.

**Figure 4:**
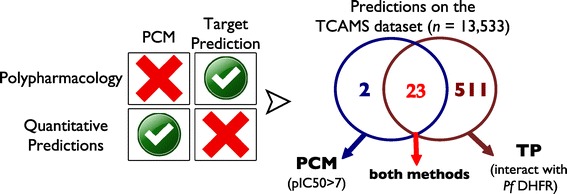
Complementarity between *in silico* target prediction and PCM. The target prediction algorithm predicted 534 compounds of the GSK TCAMS dataset to interact with DHFR, representing 3.95% of the total number of compounds in this dataset. Out of these 534 compounds, the PCM model predicted 23 compounds to have a pIC_50_ value of 7 or greater. Therefore, the combination of both methods permits the assessment of compound polypharmacology and provides quantitative bioactivity predictions.

In total, 534 compounds of the GSK TCAMS dataset were predicted to interact with DHFR, representing 3.95% of the total number of compounds in this dataset. Out of these 534 compounds, the predicted pIC_50_ values using PCM was 7 or greater for 25 compounds, between 6 and 7 for 92 compounds, and between 5 and 6 for 420. None of the 534 compounds was predicted to be inactive on DHFR (Figure 4). Given that many of the compounds in ChEMBL are active in the low micromolar range, it is thus not surprising to obtain most of the predictions in this range [54].

**Figure 5:**
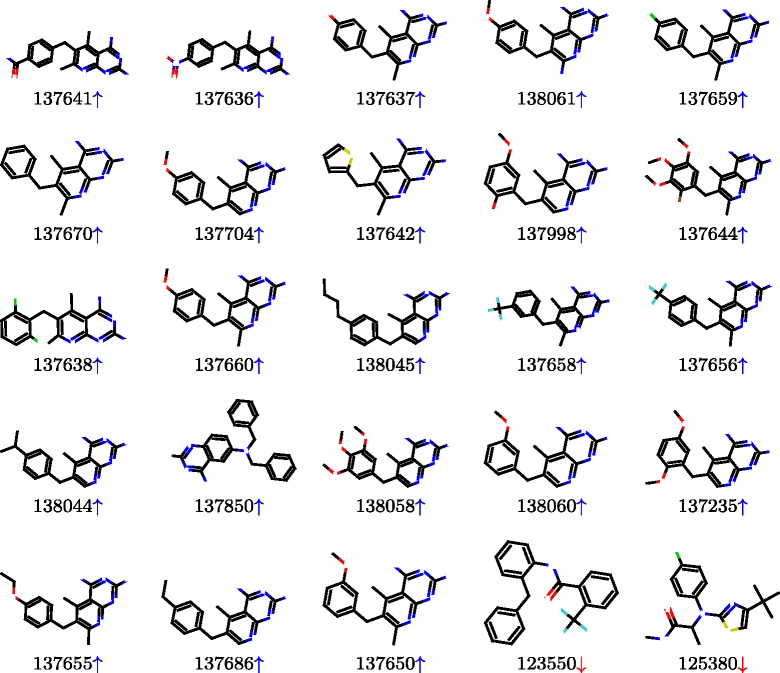
Compounds predicted to interact with DHFR by the target prediction algorithm, and predicted by the PCM model to have a pIC_50_ value higher than 7 pIC_50_ units. Compound IDs correspond to the TCMDC identifier given in the original dataset. The 23 compounds for which the IDs are accompanied by an upward-pointing arrow were identified by the two methods. The two compounds predicted to have a pIC_50_ value higher than 7 by the PCM model, but not predicted to interact with DHFR by the target prediction algorithm, are accompanied by a downward-pointing arrow. The 23 compounds predicted to be high-affinity DHFR inhibitors (upward-pointing arrows) share a common scaffold: a 5-methylpyrido[2,3-d]pyrimidine-2,4-diamine ring with an aryl substituent in the 6-position. Overall, it can be seen that these data indicate a high agreement between the target prediction algorithm and the PCM model to identify high-affinity DHFR inhibitors.

Interestingly, 23 of the 25 compounds with a predicted pIC_50_ value higher than 7 were already predicted to interact with DHFR by the target prediction algorithm (Figure 4) at the exclusion of any other target. The analysis of chemical scaffolds in the 25 compounds shows that only 2 scaffolds were identified, as 22 out of the 25 compounds (Figure 5 - excluding compounds 137850, 123550 and 125380), share a common scaffold, namely: a 5-methylpyrido[2,3-d]pyrimidine-2,4-diamine ring with an aryl substituent in the 6-position. A methyl group or an amine group in the 7-position are also present in some compounds, such as 137637 and 138061, respectively. In all compounds with the common scaffold the aryl substituent is a phenyl ring with different substituents in the 3,4,5-positions*, e.g.* methoxy, hydroxy and carboxamide, except for compound 137642, which has 2-methyl-thiophene as aryl substituent.

Two additional compounds, 123550 and 125380 (Figure 5), predicted by PCM to display pIC_50_ values of 7.11 and 7.07, respectively, represent new scaffolds. Remarkably, these two scaffolds were neither present in the PCM nor in the target prediction training set. Taken together, our results indicate a high agreement between the target prediction algorithm and the PCM model to identify high-affinity DHFR inhibitors. Using both methods simultaneously, it is possible to give higher priority to the compounds that are identified by both methods.

## Conclusions

In this study, the complementarity of *in silico* target predictions and proteochemometric modelling (PCM) was evaluated for the retrospective identification of *P. falciparum* DHFR inhibitors. The target prediction algorithm exhibited respective recall and precision values of 79% and 100% for plasmodial DHFR. The high precision value is explained by the structural similarity of plasmodial and the *T. gondii* DHFR inhibitors, which were part of the training set and were found to be relevant for extrapolation (the average MOLPRINT2D pairwise similarity between the *T. gondii* inhibitors and the plasmodial inhibitors was 16%, whereas the average pairwise similarity within the plasmodial dataset and the *T. gondii* dataset was 19% and 18% respectively). We showed that high-affinity inhibitors from the GSK TCAMS phenotypic dataset are independently identified by both methods: 534 compounds from the GSK TCAMS dataset were identified as DHFR inhibitors by the target prediction algorithm, whereas the PCM algorithm identified 25 high affinity compounds, 23 of which were already identified by the target prediction algorithm. The combination of both methods permits the assessment of compound polypharmacology and provides insight into the potency/affinity of small molecules.

We presented an approach that can be potentially extended to other human, bacterial or plasmodial targets. The inherent capability of PCM to combine bioactivity data for related targets, even for targets spanning distant phyla, is likely to improve the mining of currently available multi-target bioactivity databases. Similarly, domain-based extrapolation permits *in silico* target predictions to be extended to non-mammalian orthologous proteins for which less bioactivity data is usually available.

## Additional files

Additional file 1:
**Training set for the target prediction algorithm.**
Additional file 2:
**InterPro domain annotations for 148** ***Plasmodium falciparum*** **targets.**
Additional file 3:
**Supplementary Figures and Supplementary Table S3.**
Additional file 4:
**Supplementary Figures and Supplementary Table S3.**
