# Supplementary material for: Proteochemometric modelling coupled to in silico target prediction: an integrated approach for the simultaneous prediction of polypharmacology and binding affinity/potency of small molecules
Source: J Cheminform. 2015 Apr 15;7:15. doi: 10.1186/s13321-015-0063-9 (PMC4413554; doi:10.1186/s13321-015-0063-9)
Supplement: Additional file 3: — Supplementary Figures and Supplementary Table S3. [file 13321_2015_63_MOESM3_ESM.pdf]

Proteochemometric modelling coupled to *in silico* target prediction: an integrated approach for the simultaneous prediction of polypharmacology and binding affinity of small molecules

Shardul Paricharak,[1,2,†] Isidro Cortés-Ciriano,[3,†] Adriaan P. IJzerman,[2] Thérèse E. Malliavin[3,*] and Andreas Bender[1,*]

[1] Unilever Centre for Molecular Science Informatics, Department of Chemistry, University of Cambridge, Lensfield Road, CB2 1EW, Cambridge

[2] Division of Medicinal Chemistry, Leiden Academic Centre for Drug Research, Leiden University, P.O. Box 9502, 2300 RA Leiden, The Netherlands

[3] Unité de Bioinformatique Structurale, Institut Pasteur and CNRS UMR 3825, Structural Biology and Chemistry Department, 25-28, rue du Dr. Roux, 75 724 Paris, France

† Authors contributed equally to this work

*To whom correspondence should be addressed.

Supplementary Information

Supplementary Figures and Tables.

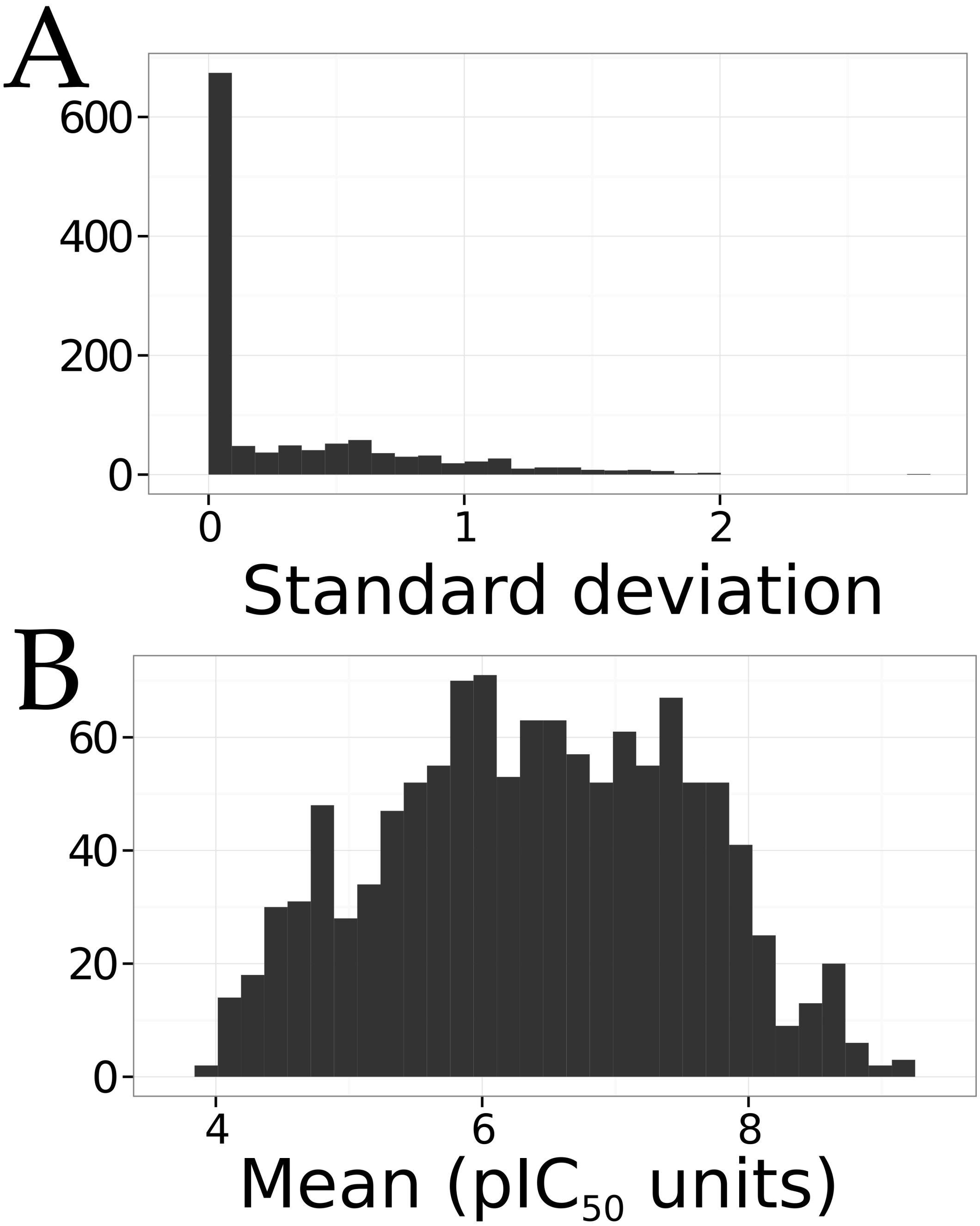

Supplementary Figure S1. A. Distribution of the standard deviation of the repeated bioactivity values in the PCM dataset. More than 90% of the bioactivity values corresponding to compound-target combinations annotated with more than one pIC_{50} value exhibited a standard deviation below 1 pIC_{50} unit,

and presented a skewed distribution towards zero. **B. Distribution of mean pIC_{50} values for the compound-target combinations annotated with more than one bioactivity value.** Mean pIC_{50} values for those compound-target combinations annotated with more than one bioactivity value span the whole bioactivity range considered in this study. On the basis of these distributions, the mean of the repeated measurements was taken as the bioactivity value.

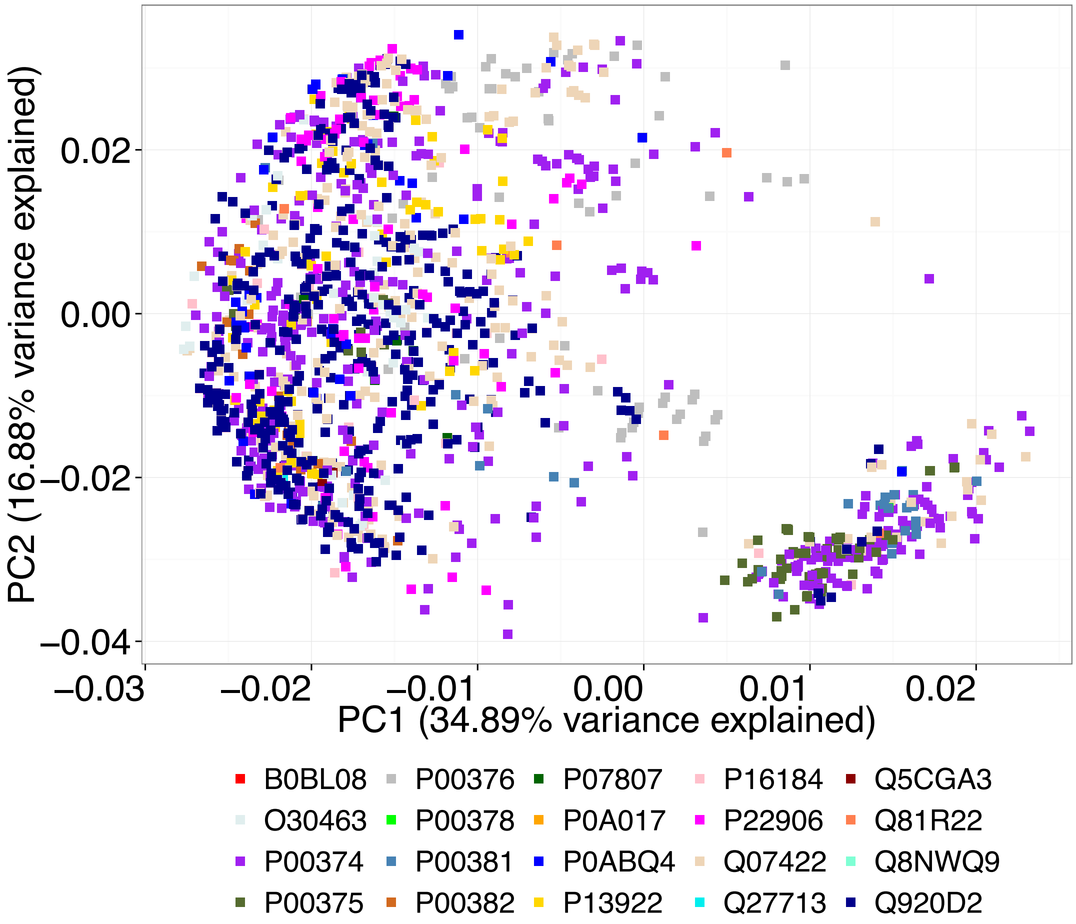

Supplementary Figure S2. PCA of the compounds present in the PCM dataset. The first two principal components explained 51.77% of the variance, calculated on the pairwise Spearman's rank correlation matrix using the same compound descriptors that served to train the models. Overall, compounds are overall structurally similar across targets, as clusters are composed of compounds with bioactivities annotated against several targets. Therefore, the prediction of compound bioactivities by PCM will mainly rely on an appropriate description of the different DHFR binding sites.

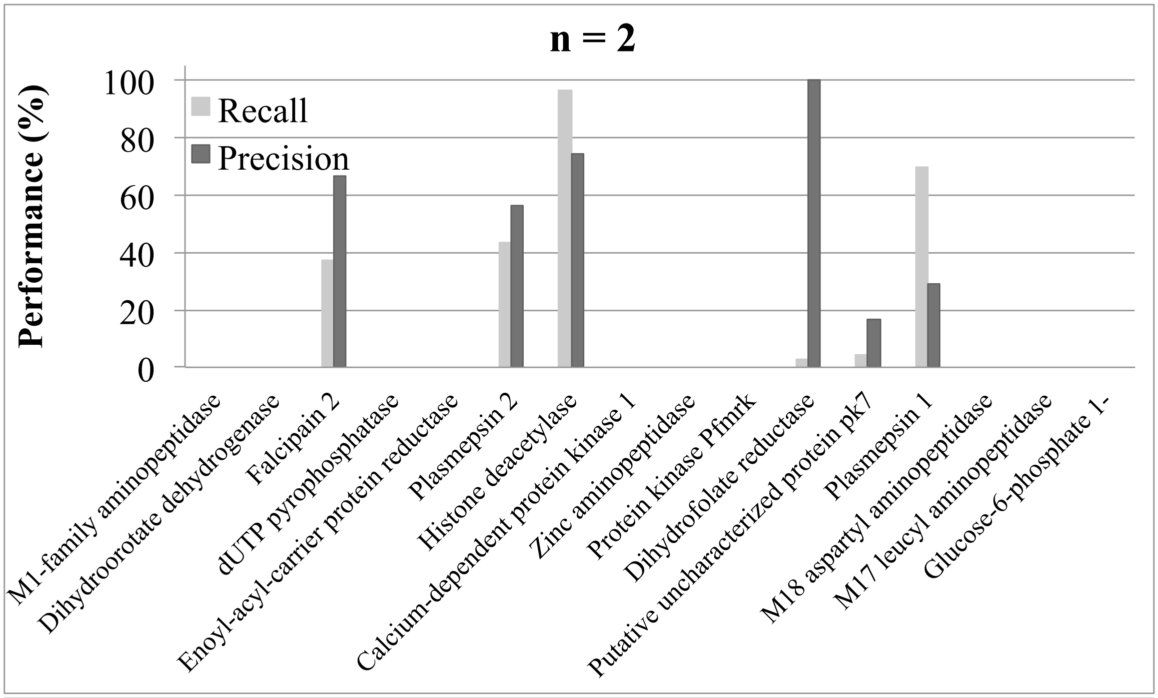
n = 2
Performance (%)
100
80
60
40
20
0
Recall
Precision
M1-family aminopeptidase
Dihydroorotate dehydrogenase
Falcipain 2
dUTP pyrophosphatase
Enoyl-acyl-carrier protein reductase
Plasmepsin 2
Histone deacetylase
Calcium-dependent protein kinase 1
Zinc aminopeptidase
Protein kinase Pfmrk
Dihydrofolate reductase
Putative uncharacterized protein pk7
Plasmepsin 1
M18 aspartyl aminopeptidase
M17 leucyl aminopeptidase
Glucose-6-phosphate 1-

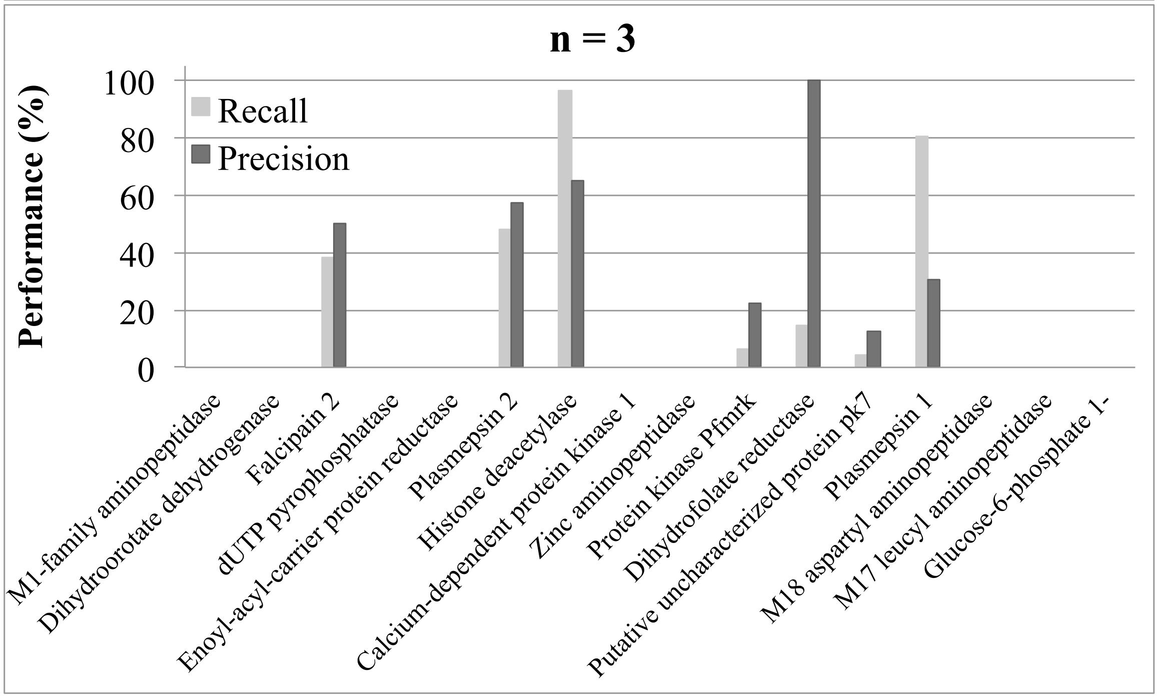
n = 3
Performance (%)
100
80
60
40
20
0
Recall
Precision
M1-family aminopeptidase
Dihydroorotate dehydrogenase
Falcipain 2
dUTP pyrophosphatase
Enoyl-acyl-carrier protein reductase
Plasmepsin 2
Histone deacetylase
Calcium-dependent protein kinase 1
Zinc aminopeptidase
Protein kinase Pfmrk
Dihydrofolate reductase
Putative uncharacterized protein pk7
Plasmepsin 1
M18 aspartyl aminopeptidase
M17 leucyl aminopeptidase
Glucose-6-phosphate 1-

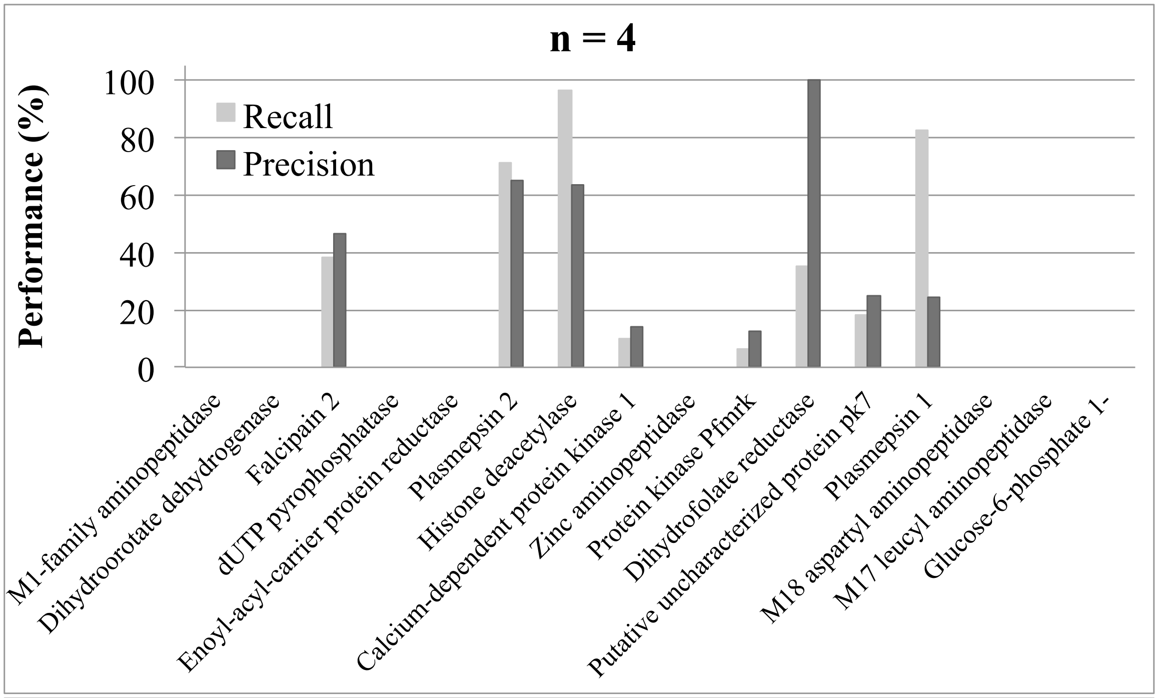

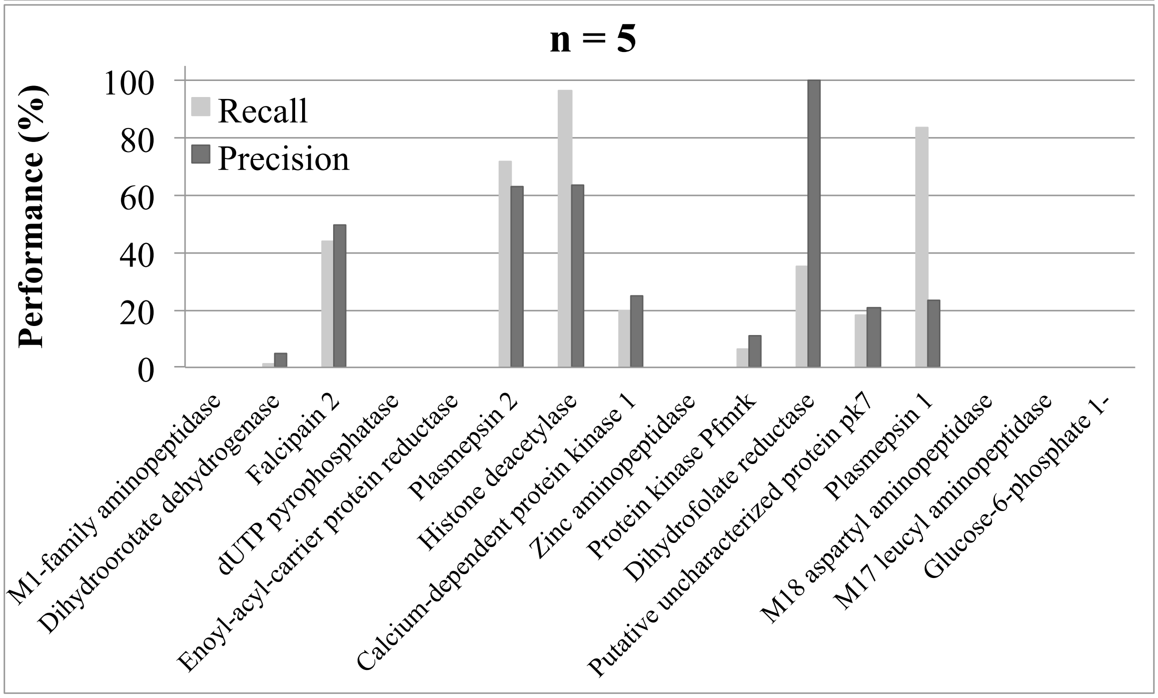

Supplementary Figure S3. Performance of the target prediction model per protein class for 16 annotated plasmodial protein targets for which the number of associated instances found in ChEMBL is 20 or greater, with a Tanimoto similarity cut-off of 0.5. The performance varies widely across target classes. For the majority of targets (all aminopeptidases, calcium-dependent protein kinase 1, protein kinase Pfmrk, glucose-6-phosphate-1-dehydrogenase, dihydroorotate dehydrogenase, dUTP

pyrophosphatase and enoyl-acyl-carrier protein reductase), performance is low, with both recall and precision values below 30%. However, for a number of targets, the performance is much higher (with recall values up to ~60% and precision values up to 100%). Further investigation revealed that the targets for which the prediction algorithm performed well (plasmepsin 1 and 2, histone deacetylase, DHFR and to a lesser extent, falcipain 2) were plasmodial orthologs of non-plasmodial protein targets.

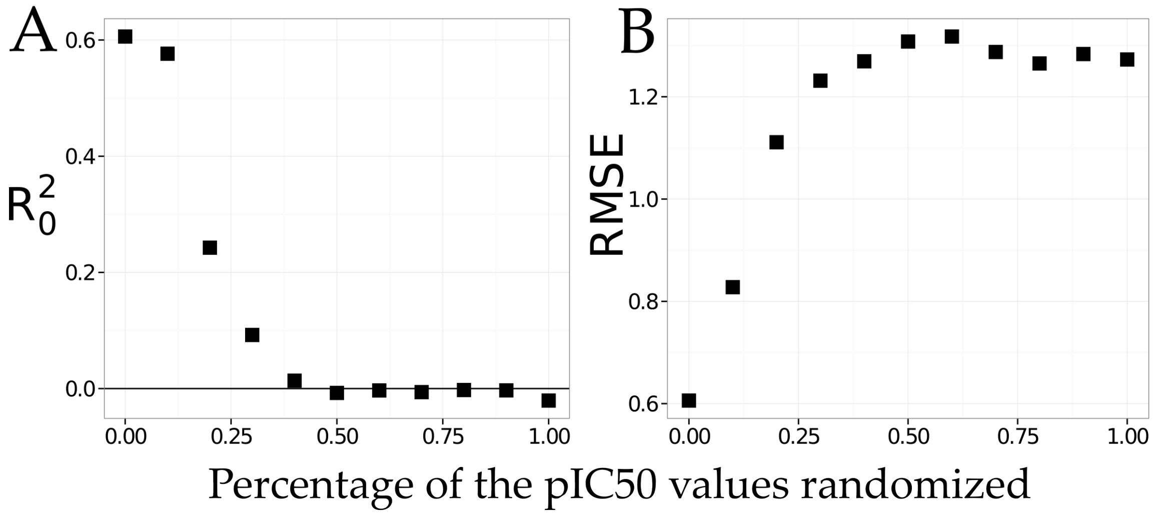

Supplementary Figure S4. **Y-scrambling validation of the PCM model.** Evolution of model performance, quantified by (**A**) $R^2_{0\ test}$ and (**B**) $RMSE_{ext}$, as a function of the percentage of the pIC_{50} values randomized. $R^2_{0\ test}$ values become zero or negative when 40% of the pIC_{50} values are randomized, thus indicating that the structure-activity relationships established by the PCM model does not stem from chance correlations.

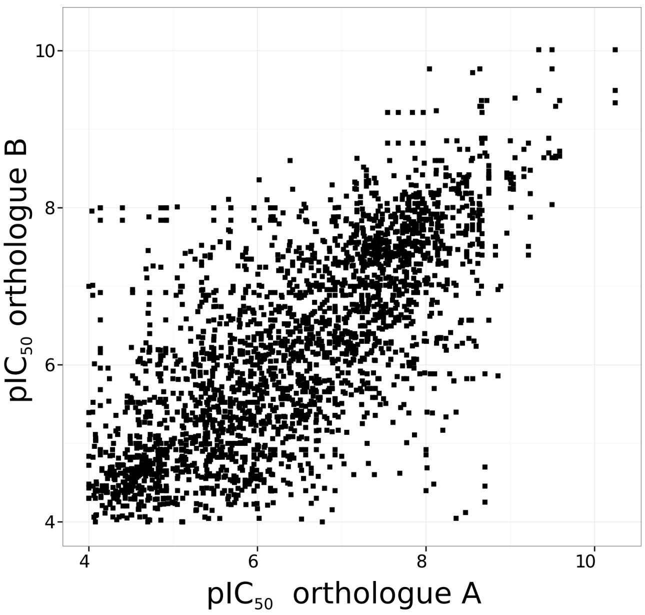

Supplementary Figure S5. Correlation between the experimental pIC_{50} values of identical compounds present in the PCM dataset against orthologous DHFR sequences. All combinations of two elements, referred to in the plot as A and B, were generated for those compounds for which pIC_{50} values on more than two DHFR sequences were available. Overall, a high correlation can be observed for compound bioactivities on orthologous sequences, which was also illustrated by the high performance of the Family QSAR model.

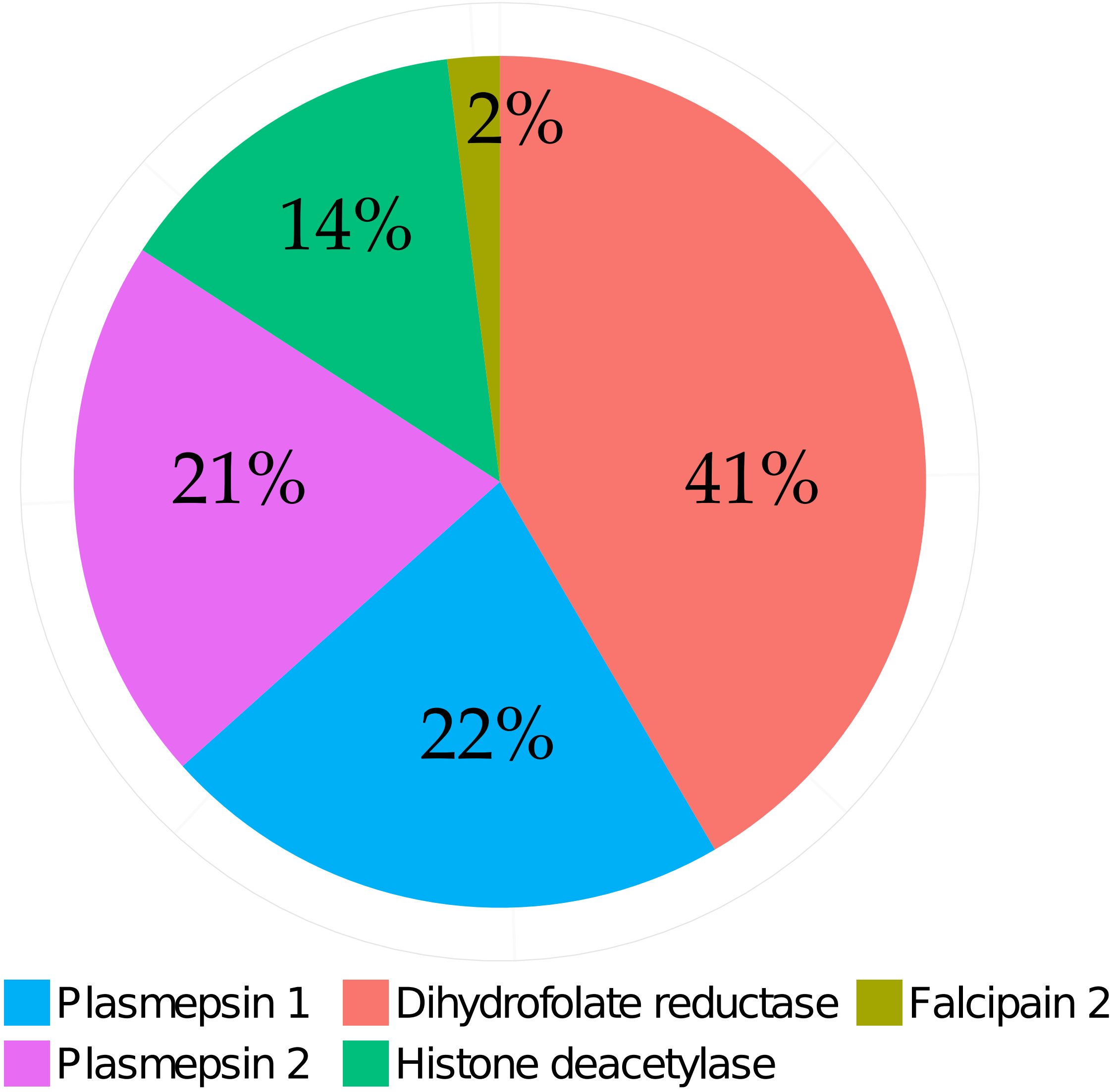

Supplementary Figure S6. Distribution of predicted plasmodial targets in the GSK TCAMS dataset. Predictions for 5 proteins for which the target prediction exhibited an F-measure value > 40% were considered, resulting in a total of 1,291 predictions for 1,017 compounds. DHFR is the most commonly predicted target, which represents 41% of the total predictions. All targets predicted have previously been proposed as targets for anti-malarial therapy (see main text).

Table S1 and S2 are given in two additional files.

Table S3. Composition of the PCM dataset. A total of 20 eukaryotic, protozoan and bacterial DHFR sequences were considered in this study. Rat and human DHFR are annotated with 759 and 753 pIC_{50} values respectively, followed by *Toxoplasma gondii* DHFR, with 532 annotations.

ChEMBL ID	UniProt ID	Organism	Number of data points
CHEMBL2363	Q920D2	*Rattus norvegicus*	759
CHEMBL3963	Q27713	*Plasmodium berghei str. ANKA*	4
CHEMBL2627	P00382	*Escherichia coli*	12
CHEMBL3644	Q8NWQ9	*Staphylococcus aureus subsp. Aureus MW2*	11
CHEMBL5457	O30463	*Mycobacterium avium*	59
CHEMBL2425	Q07422	*Toxoplasma gondii*	532
CHEMBL1809	P0ABQ4	*Escherichia coli K-12*	93
CHEMBL1926	P16184	*Pneumocystis carinii*	371
CHEMBL5441	B0BL08	*Escherichia coli*	18
CHEMBL1075051	P00376	*Bos taurus*	79
CHEMBL2902	P00381	*Lactobacillus casei*	107
CHEMBL1681620	P0A017	*Staphylococcus aureus*	6
CHEMBL2576	P07807	*Saccharomyces cerevisiae S288c*	47
CHEMBL2329	P22906	*Candida albicans*	134
CHEMBL202	P00374	*Homo sapiens*	753
CHEMBL3327	Q5CGA3	*Cryptosporidium hominis*	4

CHEMBL4564	P00375	*Mus musculus*	67
CHEMBL5270	Q81R22	*Bacillus anthracis*	20
CHEMBL2575	P00378	*Gallus gallus*	7
CHEMBL1939	P13922	*Plasmodium falciparum eK1*	73
